# WOMEN AND MEN PROFIT EQUALLY FROM CARDIAC REHABILITATION: A SECONDARY ANALYSIS OF THE OPTICARE RCT

**DOI:** 10.2340/jrm.v58.44504

**Published:** 2026-01-07

**Authors:** Nienke TER HOEVE, Marie DE BAKKER, Madoka SUNAMURA, Jeanine E. ROETERS VAN LENNEP, Eric BOERSMA, Rita J.G. VAN DEN BERG-EMONS

**Affiliations:** 1 Capri Cardiac Rehabilitation, Rotterdam; 2 Erasmus MC University Medical Centre, Department of Rehabilitation Medicine, Rotterdam; 3 Erasmus MC University Medical Centre, Department of Cardiology, Rotterdam; 4 Franciscus Gasthuis & Vlietland, Department of Cardiology, Rotterdam; 5 Erasmus MC University Medical Centre, Department of Internal Medicine, Rotterdam, The Netherlands

**Keywords:** cardiovascular disease, cardiac rehabilitation, sex differences, lifestyle, psychosocial well-being, cardiovascular risk factors, aerobic capacity

## Abstract

**Purpose:**

To explore sex-specific differences in cardiac rehabilitation (CR) outcomes.

**Methods:**

Aerobic capacity (6-Minute Walk Test), physical behaviour (accelerometer), cardiovascular risk profile (weight, blood pressure, cholesterol), and psychosocial well-being (questionnaires) were measured in patients after an acute coronary syndrome (147 women, 642 men) at CR start and completion, and 18 months’ follow-up. Sex differences were studied using GEE models adjusted for age and differences in baseline characteristics. Additionally, whether men and women met targets associated with health risk reductions was assessed.

**Results:**

Both sexes experienced similar CR benefits. Only for depressive symptoms did women show larger improvements (HADS score; ♀: –2.7 vs ♂: –1.1; *p* = 0.017). Nevertheless, long-term follow-up revealed women still lagged in meeting targets for physical activity (♀: 76.5% vs ♂: 93.1%; *p* < 0.001) and anxiety symptoms (♀: 75.5% vs ♂: 86.8%; *p* < 0.001), while men lagged in meeting aerobic capacity targets (♀: 71.3% vs ♂: 58.8%; *p* < 0.001).

**Conclusion:**

Women experience similar CR benefits to men in aerobic capacity, physical behaviour, cardiovascular risk profile, anxiety, and quality of life, with greater improvement in depressive symptoms. However, target values were less often met by women in physical behaviour and psychosocial well-being, and by men in aerobic capacity. Tailored CR programmes may be needed to address the unique needs of women and men.

## LAY ABSTRACT

This study investigated whether women and men benefit equally from cardiac rehabilitation. We followed 147 women and 642 men who participated in cardiac rehabilitation, measuring their aerobic fitness, cardiovascular health, and mental well-being at the start, end, and 18 months after the programme. Overall, both women and men showed similar improvements, with women experiencing greater improvements in depressive symptoms. Nevertheless, women continued to have more difficulties in meeting physical activity goals and were more affected by anxiety symptoms, while men were more often not meeting their aerobic fitness targets. These findings suggest that cardiac rehabilitation programmes may need to be adjusted to better address the specific needs of both women and men.

Cardiac rehabilitation (CR) is essential for secondary prevention in patients with coronary heart disease (CHD) (1). For decades, CHD has been seen as a men’s disease. Nevertheless, CHD mortality is comparable between the 2 sexes, with 20% of women and 19% of men dying of CHD (2). Contrary to this, women are less likely to participate in CR and the inclusion of women in clinical CR trials has only been between 12% and 26% (3, 4). Nevertheless, outcomes of these studies have been generalized to both sexes. As a result, CR guidelines are based on research performed in populations mainly consisting of men.

Sex-specific CR guidelines might be needed, as it is well known that differences exist between women and men. Previous research for instance has shown that women with CHD are more likely to be obese and are less likely to reach target levels for cholesterol and physical behaviour than men (5). In addition, women who enter CR have less optimal psychosocial health (6, 7). Although sex differences at the start of CR have been well studied, much less has been reported on sex differences in CR outcomes. Results of a limited number of studies suggest that women show equal improvements in cardio-metabolic outcomes (8, 9) and psychosocial health (6, 9, 10), while improvements in aerobic capacity (11, 12) and quality of life (9) are lower than in men. Findings from these previous studies are, however, limited as few (non-randomized) studies have been performed on a small set of outcomes. Furthermore, most protocols were designed to evaluate short-term effectiveness only. Because CR is a multidisciplinary intervention that focuses on long-term improvement in cardiovascular and psychosocial health, more comprehensive and long-term research on sex differences in a broader range of CR outcomes is warranted.

The purpose of the current study was to investigate whether differences exist in improvements during and after CR between women and men diagnosed with acute coronary syndrome (ACS) regarding aerobic capacity, physical behaviour, cardiovascular risk profile, and psychosocial well-being. To investigate this, data from the OPTICARE RCT, in which 914 participants (of which 175 women) with ACS were enrolled (13), is used. The OPTICARE study was originally designed to compare the effects of 2 advanced and extended CR programmes with standard CR (13–15).

## MATERIALS AND METHODS

### Patient population

This study concerns a secondary analysis of data from the OPTICARE RCT. A total of 914 patients with documented ACS were included. Exclusion criteria were severe physical and/or cognitive impairments that could limit CR participation, age < 18 years and non-proficiency in Dutch. All patients provided written informed consent, and the study was approved by the Medical Ethics Committee of the Erasmus MC in Rotterdam, the Netherlands (MEC-2010-391). The study was prospectively registered at clinicaltrials.gov (NCT01395095).

### CR programme

Patients who participated in the OPTICARE RCT were randomized in a 1:1:1 ratio to standard CR (CR-only), standard CR extended with additional behavioural face-to-face group counselling (CR+F), or standard CR extended with additional behavioural individual telephonic counselling (CR+T). The standard CR was in line with European guidelines (16), lasted 3 months and comprised 2 x 75-min exercise sessions per week. Additionally, educational sessions were offered covering medical information and information on a heart-healthy lifestyle and coping with emotions. Furthermore, patients had the option to participate in group counselling sessions on stress management, smoking cessation, and healthy diet or received individual counselling with a social worker, dietitian, or psychologist/psychiatrist. Patients randomized to the CR+F group received 3 additional behavioural face-to-face group counselling sessions on physical activity during the 3-month CR programme. Moreover, they participated in an after-care programme for 9 months consisting of 3 face-to-face group counselling sessions focusing on adopting a healthy lifestyle plus psychosocial well-being. Patients randomized to the CR+T programme participated in an additional 9-month after-care programme consisting of 5–6 individual behavioural counselling sessions by telephone, which also focused on healthy lifestyle and psychosocial well-being. Further details on the interventions have been published previously (14). Outcomes of the OPTICARE RCT revealed that patients randomized to CR+F showed greater improvements in physical activity and aerobic capacity compared with standard CR in the short term; however, these improvements did not persist in the long term. On other outcomes, there were no between-group differences. Patients randomized to CR+T did not experience additional benefits (13–15).

As we were interested in sex differences in effectiveness of CR, we included only the 771 patients (84% of total database) who completed CR. To assess whether this selection could introduce potential bias, we analysed whether completion rates differed between women and men. In line with previous studies, completion of CR was defined as participating in at least 75% of the standard CR sessions (13). Moreover, all included patients were analysed together regardless of allocated treatment, as our main aim was to investigate the differences between women and men in outcomes of guideline-based CR. Nevertheless, a secondary analysis was performed to study whether sex differences might vary across the CR types.

### Measurements and procedure

Patient characteristics were collected at the start of CR (pre-CR). Outcomes regarding aerobic capacity, physical behaviour, cardiovascular risk profile, and psychosocial well-being were all collected at CR start (pre-CR), at CR completion (post-CR), and 18 months after CR start (long term).

*Patient characteristics*. Age, sex, referral diagnosis and medical treatment, history of cardiovascular disease, cardiovascular risk factors, and medication details were collected from the hospital discharge letter. Educational level, marital status, and employment were collected with a self-designed questionnaire. Participation in different components of the CR programme (e.g., educational sessions or counselling) was extracted from CR patient files.

*Aerobic capacity and physical behaviour*. Aerobic capacity was measured with a 6-Minute Walk Test (6MWT) according to the American Thoracic Society guidelines (17). Patients were asked to walk back and forth along a 30-m-long circuit. Standardized encouragement was given every minute. In addition to the distance walked, we calculated whether patients met sex-specific reference values based on age, height, and weight (18). Physical behaviour (physical activity and sedentary behaviour) (19) was measured with ActiGraph GT3X accelerometers (LLC, Pensacola, FL, USA) for a maximum of 8 days at each measurement occasion during waking hours. Data were only included in analyses if the accelerometer was worn for a minimum of 10 h during at least 4 days. Data were sampled at 30 Hz. ActiLife software (version 6.6.0, LLC, Pensacola, FL, USA) was used to convert acceleration into daily steps and activity counts (using the vector magnitude, a composite measure of accelerations on the x, y, and z axes). Activity counts were summed over 15-s epochs and categorized as moderate-to-vigorous physical activity (≥ 672.5 counts per 15 s epoch); light activity (>375.5 and < 672.5 counts per 15 s epoch) and sedentary behaviour (≤ 37.5 counts per 15 s epoch). Duration of time spent in each category was expressed as percentage of wear time. Additionally, we calculated the number of patients meeting (non-sex-specific) health targets, which have been associated with substantial risk reductions in incident CVD (≥ 7,126 steps) (20) or premature mortality (≥ 24 min of moderate to vigorous activity, ≥ 375 min of light physical activity, < 9.5 h of sedentary behaviour) (21). Further details have been published previously (15).

*Cardiovascular risk profile*. Body mass index (BMI) was calculated from weight and height (kg/m^2^). Systolic blood pressure was measured using a validated sphygmomanometer. Fasting blood samples were used to determine LDL cholesterol (mmol/L) and HDL cholesterol (mmol/L). Smoking status was determined using a carbon monoxide breath analyser (piCO+ Smokerlyzer, Bedfont Scientific, Maidstone, UK). Additionally, we assessed whether patients met non-sex-specific health target values aimed at reducing the risk of secondary cardiovascular events. These targets were based on the guidelines in effect during the study period, as defined by the European Society of Cardiology (ESC) for LDL (≤ 1.8 mmol/L) and blood pressure (≤ 140 mmHg) (22), and by the World Health Organization (WHO) for BMI (≤ 25 kg/m²) (23). Even though guidelines defining a specific target for HDL are lacking, we have set the target at ≥ 1.0 mmol/L, as higher levels of HDL cholesterol are generally associated with reduced cardiovascular risk.

*Psychosocial well-being*. Symptoms of anxiety and depression were measured with the Hospital Anxiety and Depression Scale (HADS) (24). The HADS has a subscale for anxiety symptoms (HADS-A) and a subscale for depressive symptoms (HADS-D), both with continuous scores between 0 and 21, with higher scores indicating more anxiety and depressive symptoms. A score below 8 on each subscale was considered the threshold for meeting the (non-sex-specific) target for both anxiety and depressive symptoms (24). Satisfaction with participation in society was measured with a subscale of the Utrecht Scale for Evaluating Rehabilitation-Participation (USER-P) (25). The outcome of this subscale is a continuous score between 0 and 100, with higher scores indicating higher satisfaction. Health-related quality of life (HRQoL) was measured with the MacNew questionnaire (26). Outcome is a score between 1 and 7 with a higher score indicating a higher HRQoL. No target values are available for either satisfaction with participation in society or HRQoL.

### Statistical analysis

Descriptive statistics were used to present baseline characteristics. Differences in characteristics between patients included and excluded for analysis and between women and men were examined using Student’s *t*-tests (continuous data) or χ^2^ tests (categorical data).

To investigate differences in CR outcomes between sexes, we used a generalized estimating equation (GEE) approach, as it corrects for the dependency of repeated observations within 1 individual. A separate model was created for each CR outcome. We added to the model sex and the different time points as dummy variables to evaluate baseline differences between women and men and mean changes between time points. Furthermore, an interaction term between the dummy time variables and sex was included to evaluate differences in mean changes over time between women and men. As women typically experience cardiac events at a later age, which may influence CR participation and our outcomes of interest, we included age, along with other patient characteristics that were significantly different between women and men at baseline, as potential confounder in the models. Missing baseline characteristics were handled by multiple imputations. Endpoints (CR outcomes) were not imputed, as GEE models utilize all available data on the dependent outcome, rather than only complete cases.

Two additional secondary analyses were performed. First, to gain insight into the clinical relevance of changes seen during CR and the differences in these changes between men and women, we assessed differences in meeting health target values for the defined CR outcomes at each time point using χ^2^ tests. For outcomes with expected cell counts less than 5, Fisher’s exact tests were applied. Second, to explore whether sex differences varied according to the allocated CR programme (CR-only, CR+F, CR+T), generalized linear models were performed for the 3 intervention groups separately and outcomes were compared between intervention groups using a Z-test. The difference in mean change between women and men in the CR-only group was compared with the difference in mean change between women and men in the CR+F and the CR+T group.

For analysis of baseline characteristics SPSS version 26 was used (IBM Corp, Armonk, NY, USA). Missing data were handled using the MICE package in R (version 3.6.2; R Foundation for Statistical Computing, Vienna, Austria) and the GEEPACK package was used to fit the marginal models using a GEE approach. For all analyses, a two-sided *p*-value of < 0.05 was considered significant.

## RESULTS

### Study population

A total of 771 (84%) patients completed CR and were included for analysis. Completion rates did not differ between women and men (♀: 84.0% vs ♂:84.4%, *p* = 0.886). The included study population consisted of 147 women (mean age: 57.8 years) and 624 men (57.0 years, *p*-value ♀ vs ♂ = 0.310). Women less often had a partner (♀: 71.9% vs ♂: 84.9%, *p* = 0.001), were less often employed (♀: 51.3% vs ♂: 62.7%, *p* = 0.026), and less often were overweight (♀: 69.4% vs ♂: 78.8%, *p* = 0.015) compared with men. Furthermore, women were less often treated with revascularization (♀: 86.4% vs ♂: 93.3%, *p*< 0.001), more often used thienopyridines (♀: 92.5% vs ♂: 81.6%, *p* = 0.001, and less often used ACE inhibitors (♀: 63.3% vs ♂: 72.8%, *p* = 0.022). Other baseline characteristics did not differ between women and men (Table I). The amount of missing data in defined outcomes at baseline and follow-up did not differ between women and men, except for HDL at CR start, which was more frequently missing in women (♀: 8.8% vs ♂: 4.5%, *p* = 0.034) Detailed information on missing data for each outcome can be found in Appendix S1).

**Table I:** Baseline characteristics of study population (*n* = 771)

Characteristics	Women ♀ (*n* = 147)	Men ♂ (*n* = 624)	*p*-value ♂ vs ♀ ^*^
Age, mean years±SD	57.8±9.3	57.0±9.1	0.310
Educational level, %^1^			0.124
• Low	7.6	4.1	
• Intermediate	68.0	64.5	
• High	24.4	31.4	
Partnered, %^2^	71.9	84.9	**0.001**
Employed, %^3^	51.3	62.7	**0.026**
Medical treatment, %			**< 0.001**
• No revascularisation	13.6	6.7	
• PCI	81.6	76.6	
• CABG	4.8	16.7	
No cardiac history, %	86.4	84.5	0.555
Risk factors, %			
• Family history	56.5	52.4	0.375
• Diabetes	15.6	12.7	0.336
• Dyslipidaemia	32.0	35.4	0.430
• Hypertension	46.9	40.1	0.128
• Smoking (pre-ACS)	34.7	41.5	0.130
• Overweight^4^	69.4	78.8	**0.015**
Cardiac medication, %^5^			
• Acetylsalicylic acids	99.3	96.6	0.077
• Thienopyridines	92.5	81.6	**0.001**
• Statins	94.6	96.9	0.158
• Beta blockers	80.3	83.3	0.390
• ACE inhibitors	63.3	72.8	**0.022**
Participation in programme^6^			
• Training sessions, number	22.9±5.8	23.2±5.8	0.581
• Educational sessions, %	78.1	75.4	0.496
• Lifestyle counselling sessions, %			
Stress management	28.8	24.4	0.297
Smoking cessation	5.5	3.4	0.230
Healthy diet	9.6	6.6	0.206
• Individual treatment, %			
Dietitian	19.2	15.8	0.315
Social worker	18.5	9.3	**0.001**
Psychologist/psychiatry	5.5	4.2	0.492

1 Data missing for *n* = 143 (18%);

2 data missing for *n* = 141 (18%);

3 data missing for *n* = 191 (25%);

4 data missing for *n* = 1 (0.001%);

5 data missing for *n* = 3 (0.004%);

6 data missing for *n* = 3 (0.004%).

PCI: percutaneous coronary intervention; CABG: coronary artery bypass grafting; ACS: Acute coronary syndrome.

* *p*-values are based on *t*-test or X^2^-tests and reflect sex differences in baseline characterstics.

### Sex differences in CR treatment

Women more often participated in individual treatment by a social worker (♀: 18.5% vs ♂: 9.3%, *p* = 0.001). Compliance with exercise sessions, educational sessions, and lifestyle counselling was comparable between women and men (see Table I).

### CR outcomes

**Fig. 1:**
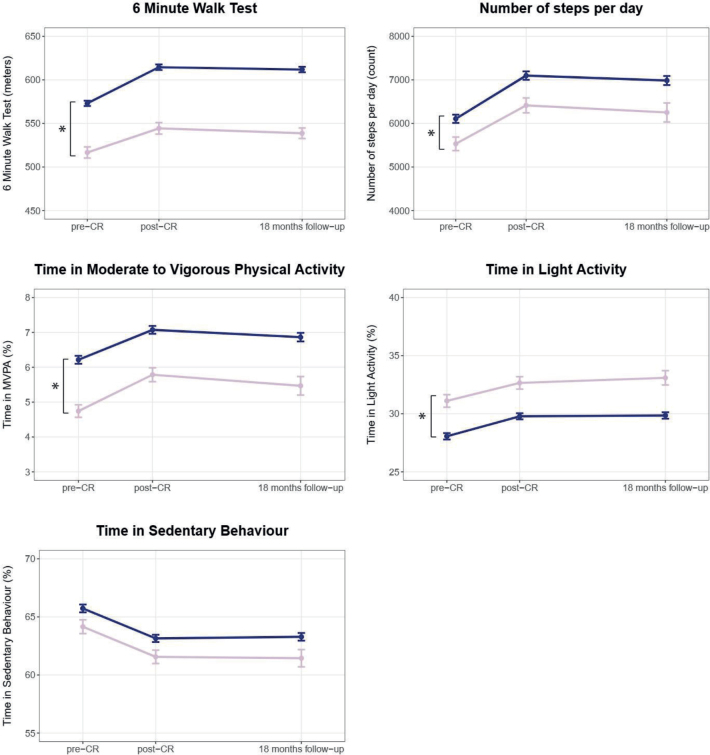
Sex differences in aerobic capacity and physical behaviour before cardiac rehabilitation, after cardiac rehabilitation, and at 18 months’ follow-up (raw data). Pink line = women; blue line = men; graphs show means and standard error of the means. CR: cardiac rehabilitation. *Significant baseline difference (vertical) or difference in mean change (horizontal) between women and men.

*Aerobic capacity and physical behaviour*. At CR start, women had lower aerobic capacity than men (6MWT distance [95% CI]: ♀: 512.0 m [443.2; 580.8] vs ♂: 571.9 m [501.8; 642.0]; *p* < 0.001). In interpreting these findings, it should be noted that, unlike other outcomes, separate health target values exist for aerobic capacity for women and men, which is addressed in the secondary analyses below. Furthermore, there was a baseline difference in physical activity with women making fewer daily steps (♀: 5,591 [3,542; 7,639] vs ♂: 6,124 [4,004; 8,244]; *p* = 0.023), spending less time in moderate-to-vigorous physical activity (♀: 4.8% [2.3; 7.3] vs ♂: 6.2% [3.6; 8.8], *p* < 0.001), which equals circa 42 min/day vs 54 min/day for a mean daily wear time of 14.6 h and spending more time in light physical activity (♀: 31.2% [24.4; 38.0] vs ♂: 28.1% [21.5; 34.8], which equals circa 273 min/day vs 246 min/day; *p* < 0.001). There were no baseline differences in sedentary behaviour. Women and men showed comparable improvements in aerobic capacity, physical activity, and sedentary behaviour during CR over time. These improvements were maintained for both sexes in the long term with no between-group differences (Fig. 1 and Appendix S2a).

**Fig. 2:**
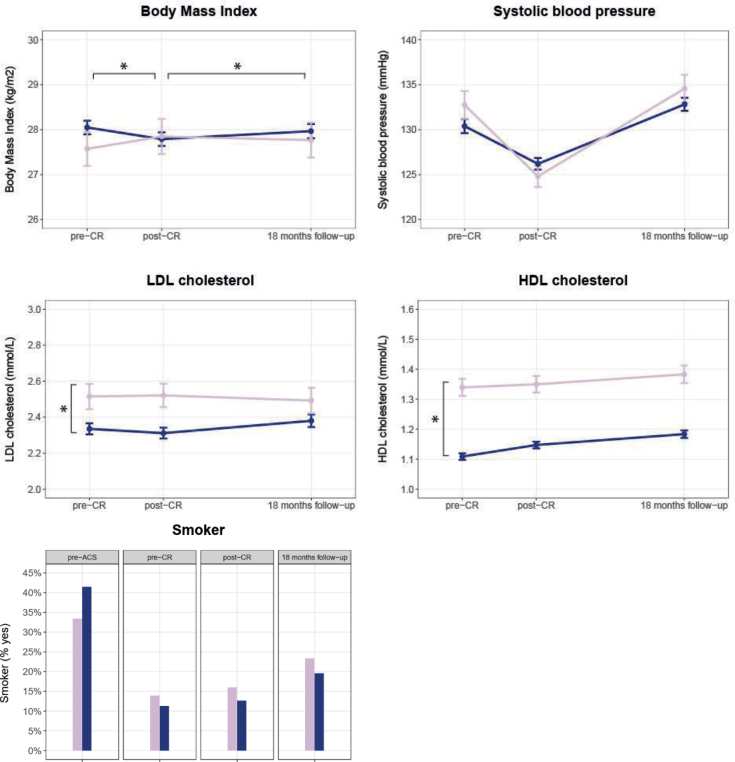
Sex differences in cardiovascular risk profile before cardiac rehabilitation, after cardiac rehabilitation, and at 18 months’ follow-up (raw data). Pink line = women; blue line = men; graphs show means and standard error of the means. CR: cardiac rehabilitation; LDL: low density lipoprotein cholesterol; HDL: high density lipoprotein cholesterol. *Significant baseline difference (vertical) or difference in mean change (horizontal) between women and men.

*Cardiovascular risk profile*. At CR start, women had higher LDL levels (mean LDL level [95% CI]: ♀: 2.5 mmol/L [1.9; 3.2] vs ♂: 2.3 mmol/L [1.7;3.0]; *p* = 0.029) and higher HDL levels (♀: 1.3 mmol/L [1.1; 1.6] vs ♂: 1.1 mmol/L [0.9; 1.4]; *p* < 0.001). Changes during and after CR in LDL and HDL were small and comparable for women and men. Although there were no baseline differences in BMI, significant differences were seen in changes between women and men during CR. During CR women increased their BMI (+0.3 [0.001; 0.7]) whereas men decreased their BMI (–0.2 [–0.3; –0.1], *p*-value ♀ vs ♂ = 0.001). After CR, the opposite was seen, with women improving their BMI (–0.1 [–0.5;0.3]) and men showing a deterioration in their BMI (+0.3 [0.2;0.5], *p*-value ♀ vs ♂ = 0.048). No sex differences were seen at baseline or during and after CR in blood pressure and smoking rate (Fig. 2 and Appendix S2b).

**Fig. 3:**
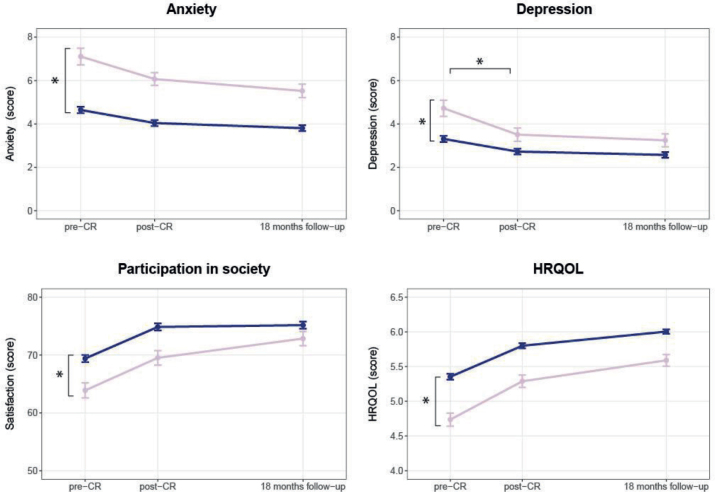
Sex differences in psychosocial well-being before cardiac rehabilitation, after cardiac rehabilitation and at 18 months follow-up (raw data). Pink line = women; blue line = men; graphs show means and standard error of the means. CR: cardiac rehabilitation; HRQoL = health-related quality of life. Anxiety and depression were measured using the Hospital Anxiety and Depressions Scale (HADS), Participation in Society using the Utrecht Scale for Evaluating Rehabilitation-Participation (USER-P), HRQoL was measured with the MacNew questionnaire. *Significant baseline difference (vertical) or difference in mean change (horizontal) between women and men.

*Psychosocial well-being*. At CR start, women had higher (worse) scores regarding anxiety (mean HADS-A score [95 CI]: ♀: 7.3 [4.0;10.6] vs ♂: 4.8 [1.5;8.1], *p* < 0.001) and depressive symptoms (HADS-D score: ♀: 4.9 [1.7;8.1] vs ♂: 3.4 [0.2;6.7], *p* = 0.001). Furthermore, women had lower (worse) scores for satisfaction with participation in society (USER-P score ♀: 63.7 [50.6;76.7] vs ♂: 68.5 [55.4; 81.6], *p* = 0.002) and HRQoL (MacNew score ♀: 4.7 [3.8; 5.6] vs ♂: 5.3 [4.4; 6.2], *p* < 0.001) compared with men. Comparable improvements were seen during and after CR in satisfaction with participation in society and HRQoL for women and men. Improvement in depressive symptoms during CR was greater for women (–1.4 [–2.0; –0.8]) compared with men (–0.6 [–0.8; –0.3], *p* = 0.009) (Fig. 3 and Appendix S2c).

### Secondary analysis

*Meeting health target values*. At CR start, women were more likely to meet health target values for aerobic capacity (♀: 56.4% vs ♂: 40.8%, *p* = 0.003), sedentary behaviour (♀: 64.3% vs ♂: 49.7%, *p* = 0.016), BMI (♀: 30.6% vs ♂: 21.2%, *p* = 0.015), and HDL (♀: 88.9% vs ♂: 66.4%, *p* < 0.001). In contrast, men were more likely to meet targets for moderate-to-vigorous physical activity (♀: 75.0% vs ♂: 88.5%, *p* = 0.001), anxiety (♀: 64.2% vs ♂: 80.9%, *p* < 0.001), and depressive symptoms (♀: 72.9% vs ♂: 88.5%, *p* < 0.001). Directly after CR, women were meeting target values more frequently for aerobic capacity (♀: 70.1% vs ♂: 58.4%, *p* = 0.033) and HDL (♀: 88.0% vs ♂: 72.4%, *p* < 0.001), while men more often met anxiety symptom targets (♀: 70.2% vs ♂: 85.5%, *p* < 0.001). At long-term follow-up, women were more likely to meet targets for aerobic capacity (♀: 71.3% vs ♂: 58.8%, *p* = 0.026), BMI (♀: 30.0% vs ♂: 21.0%, *p* = 0.035), and HDL (♀: 92.4% vs ♂: 75.4%, *p* < 0.001), while men were more likely to meet targets for moderate-to-vigorous physical activity (♀: 76.5% vs ♂: 93.1%, *p* < 0.001) and anxiety symptoms (♀: 75.5% vs ♂: 86.8%, *p* < 0.001). For further details, see Table II.

**Table II:** Cardiac rehabilitation outcomes: percentage of patients reaching health targets (%)

Factor	Pre-CR Women ♀	Men ♂	*p*-value*	Post-CR Women ♀	Men ♂	*p*-value*	18 months follow-up		
							Women ♀	Men ♂	*p*-value*
6-Minute Walk Test (m)^1^	56.4	40.8	**0.003**	70.1	58.4	**0.033**	71.3	58.8	**0.026**
Steps per day ≥ 7,126	21.4	28.5	0.185	36.5	44.3	0.220	36.8	44.0	0.278
MVPA ≥ 24 min/day	75.0	88.5	**0.001**	90.5	96.0	0.052	76.5	93.1	**< 0.001**
Light activity ≥ 375 min/day	6.0	2.9	0.161	5.4	4.3	0.681	10.3	5.8	0.186
SB < 9.5 h/day	64.3	49.7	**0.016**	71.6	53.8	0.005	64.7	51.9	0.056
Body mass index ≤ 25	30.6	21.2	**0.015**	29.7	22.7	0.081	30.0	21.0	**0.035**
Systolic BP ≤ 140 mmHG	74.1	76.3	0.587	91.2	86.7	0.145	70.2	76.2	0.160
LDL ≤ 1.8 mmol/L	25.4	27.8	0.580	22.4	28.9	0.158	22.1	27.1	0.245
HDL ≥ 1.0mmol/L	88.9	66.4	**< 0.001**	88.0	72.4	**< 0.001**	92.4	75.4	**< 0.001**
Not smoking	85.5	88.6	0.365	84.0	87.5	0.304	76.7	80.6	0.334
Anxiety symptoms < 8	64.2	80.9	**< 0.001**	70.2	85.5	**< 0.001**	75.5	86.8	**0.005**
Depressive symptoms < 8	72.9	88.5	**< 0.001**	84.6	90.8	0.061	85.7	90.7	0.142
Participation in society^2^	–	–		–	–		–	–	
HRQoL^2^	–	–		–	–		–	–	

1 Health target values for the 6-Minute Walk Test were sex-specific, whereas the health targets for other outcomes were the same for both men and women.

2 No health target (cut-off) values available for participation in society and HRQoL.

MVPA: moderate-to-vigorous physical activity; SB: sedentary behaviour; BP: blood pressure; LDL: low density lipoprotein cholesterol; HDL: high density lipoprotein cholesterol.

* *p-*values are based on X^2^-tests and reflect sex differences in percentage of patients reaching health targets at the specified timepoints.

*Sex differences according to the allocated CR programme*. To investigate whether sex differences varied according to CR programme (CR-only: 48 women, 200 men, CR+F: 54 women, 214 men, CR+T: 45 women, 210 men), we performed an exploratory analysis. Distribution of women and men did not differ between these CR programmes (*p* = 0.760). Differences in CR outcomes are presented in Appendices S3–5. Outcomes suggest that women profit less from additional face-to-face counselling (CR+F) regarding physical activity compared with men (mean change percentage of time in moderate-to-vigorous physical activity [95% CI] post-CR vs pre-CR: CR-only: ♀: +1.5% [0.8; 2.2] vs ♂: +0.5% [0.05; 1.0]; CR+F: ♀: +1.0% [0.2; 1.8] vs ♂: +1.5% [1.1; 2.0], *p* = 0.019 vs CR-only)**.** Women seem to gain additional benefit from extra face-to-face counselling regarding depressive symptoms (HADS-D score post-CR vs pre-CR: CR-only: ♀: –0.2 [–0.9; 0.5] vs ♂: –0.7 [–1.1; –0.3]; CR+F: ♀: –1.8 [–2.7; –0.9] vs ♂: –0.6 [–1.0; –0.1], *p* = 0.005 vs CR-only). Furthermore, outcomes suggest that women have less benefit from telephone counselling (CR+T) regarding smoking behaviour (odds ratio smoking post-CR vs pre-CR: CR-only: ♀: 1.0 [0.9; 1.1] vs ♂: 1.06 [1.0; 1.1]; CR+T: ♀: 1.05 [0.96; 1.15] vs ♂: 0.98 [0.95; 1.02], *p* = 0.031 vs CR-only). Women had additional benefit from telephone counselling with regard to depressive symptoms (HADS-D score post-CR vs pre-CR: CR-only: ♀: –0.2 [–0.9; 0.5] vs ♂: –0.7 [–1.1; –0.3]; CR+T: ♀: –2.4 [–3.7; –1.0] vs ♂: –0.3 [–0.7; 0.1], *p* = 0.002 vs CR-only) and HRQoL (MacNew score post-CR vs pre-CR: CR-only: ♀: +0.4 vs ♂: +0.5; CR+T: ♀: +0.8 [0.5; 1.1] vs ♂: +0.4 [0.3;0.5], *p* = 0.032 vs CR-only). Sex differences for other outcomes were comparable between the interventions. At long term follow up, there were no sex differences in effectiveness between CR programmes.

## DISCUSSION

Our study indicates that women largely experience similar benefits from CR compared with men, both immediately after CR and in the longer term (up to 18 months) regarding aerobic capacity, physical behaviour, cardiovascular risk profile, anxiety symptoms, and HRQoL. Small and clinically irrelevant sex differences were observed in weight loss, with men showing greater weight loss during CR, while women experienced more weight loss at long-term follow-up. Additionally, improvements in depressive symptoms scores during CR were more pronounced for women.

Both sexes showed clinically relevant and comparable improvements in aerobic capacity and physical behaviour during CR, with benefits being sustained up to 1.5 years. Our study aligns with previous research in cardiac patients and the general population, showing that women entering CR have a lower aerobic capacity and engage in less moderate-to-vigorous physical activity than men (5, 11, 18, 27). Regarding aerobic capacity, this difference is not resolved during CR due to the similar improvements in both groups. Nonetheless, women met sex-specific aerobic capacity targets more often than men both before and after CR, suggesting that biological factors likely contribute to the observed absolute difference. Conversely, regarding physical activity, women were meeting (non-sex specific) health targets less often at baseline. For this outcome the improvements seen during CR were slightly, though not significantly, more pronounced in women, allowing them to meet health targets as often as men by the end of CR. In the long term women were, however, again less likely to meet the health targets, indicating that they struggle to maintain these additional improvements over time. Future CR programmes may benefit from incorporating elements that specifically encourage sustained participation in physical activity for women, while focusing on further improving aerobic capacity in men. Regarding light physical activities, baseline differences were observed in absolute values, favouring women, but not in health target achievement. With regard to sedentary behaviour, there were no significant differences in absolute values, but women were meeting health targets more often. However, these sex differences were no longer present after CR, suggesting that sex-specific CR components are not needed.

At the start of CR, most cardiovascular risk factors were already well controlled in both sexes (except for LDL cholesterol), most likely due to optimal medication (13). The low proportion of patients meeting the LDL target can partly be explained by the recent change in the LDL target from 2.5 to 1.8 mmol/L, which was introduced during the study period (22). Both during CR and in the follow-up period, no sex differences were found in the minor changes seen in LDL and HDL cholesterol, blood pressure, and smoking. Nevertheless, our findings suggest that women have slightly more favourable HDL cholesterol but also slightly more unfavourable LDL cholesterol both before and after CR. These results are consistent with known sex differences in the general population, where women in their late 50s tend to have somewhat higher HDL levels as well as elevated LDL levels compared with men, the latter mainly due to the sharp increase in LDL that occurs after menopause (28). Concerning weight loss, previous studies are conflicting as to whether or not sex differences exist (29). In our study, we observed small sex differences, with men losing slightly more weight during CR, while women experienced greater weight loss in the initial months after CR. However, these differences were small, with both sexes showing changes between 0.3% and 1% of initial weight, falling well below the clinically relevant threshold of 5% (30). Although women were more likely to meet BMI targets both at baseline and after CR, approximately 70% of women and 80% of men remained overweight, and the absolute BMI differences between sexes were small. This suggests the need for additional interventions for both sexes.

The literature on sex differences regarding changes in psychosocial well-being during CR is inconsistent (9, 29, 31–33). In our study, both sexes showed improvements, with women showing even larger improvements in depressive symptoms. Improvements were sustained for both sexes after CR. The larger improvements seen in women in depressive symptoms during CR may be linked to the more frequent use of psychosocial support reported in this study, as well as their lower baseline scores, which allowed for greater improvement. Although the additional improvements allowed women to meet target values for depressive symptoms as often as men, women still met anxiety symptom targets less frequently than men after CR. The relatively high percentage of women not treated with revascularization may reflect the presence of SCAD (spontaneous coronary artery dissection) or coronary microvascular dysfunction, conditions that were less well recognized at the time this study was conducted. These patient groups are known to experience higher levels of anxiety (34), which could partly explain the observed sex differences in our outcomes. Although no target values exist for participation in society and HRQoL, the absolute differences between women and men suggest that the improvements during and after CR were insufficient to bridge the baseline gaps. The less optimal psychosocial health of women is a concern and additional attention during CR might be warranted, as it is associated with poorer outcomes after ACS (31, 35).

Overall, our study indicates that CR is equally effective for women and men. Currently, referral of women to CR is lower than for men (29). Lack of knowledge on CR benefits has been reported as a major barrier for referral (36). Hence, our study underscores the importance of ensuring that women are adequately referred for and encouraged to participate in CR. While most improvements during and after CR were similar for both sexes, health target values were less often met in physical activity and psychosocial well-being for women and in aerobic capacity for men, suggesting that tailored programmes might be needed.

Exploratory analyses as part of our study showed that men benefited more from additional face-to-face group counselling for physical activity, while women gained more from face-to-face or telephone counselling for improving psychosocial well-being. These results suggest that tailored behavioural counselling could help address psychosocial gaps seen in women, though it may not resolve disparities in physical activity. Further research into tailored CR programmes is needed. We propose that the first step in designing such programmes should involve qualitative studies to investigate the specific needs of women and men. Additionally, while our current study focused on sex differences, future research should also examine the impact of gender-related factors, such as gender identity, socioeconomic status, educational level, marital status, and work status, on CR adherence and success.

### Strengths and limitations

This study concerns a secondary analysis of data collected in the OPTICARE RCT. This database provided a unique opportunity to study sex differences in a wide range of CR outcomes. The data were collected from a large and representative group of ACS patients, increasing the generalizability of the findings. Additionally, the study explored 3 different CR interventions, allowing for a comprehensive examination of their effects on women and men. Furthermore, the patients were followed up for 1.5 years, enabling the assessment of longer-term outcomes.

In addition to these strengths, there are also limitations. One limitation is that the original study was not powered to investigate sex differences. Furthermore, due to the multidisciplinary nature of CR, we studied a large number of outcomes. As this study is meant to be exploratory, we decided to not correct for multiple testing. This could have led to some outcomes being significant due to chance. However, given that the observed outcomes align with expectations and previous research, reported differences are likely to be valid. Another limitation is that women are less likely than men to enrol in CR. Therefore, it is possible that the database primarily consists of highly motivated women. This could have led to a smaller magnitude of sex differences in outcomes. For example, we found no age difference between women and men in our study, despite the well-established fact that women tend to experience cardiac events at an older age. This discrepancy may reflect an inclusion bias, potentially underrepresenting older women. Furthermore, patients were included in the OPTICARE trial between 2011 and 2014, which could raise concerns regarding the age of the data. Nevertheless, CR has remained consistent since then and the studied programme aligns with current guidelines. One final limitation is that, except for functional capacity, all specified health targets were not sex specific. Future research is needed to more precisely define personalized target values for women and men separately.

### Conclusions

Our results suggest that women and men gain the same benefits from CR in terms of aerobic capacity, physical behaviour, cardiovascular risk factor management, anxiety symptoms, and HRQoL in the short and longer term. Improvement in depressive symptoms was even larger for women. Despite these favourable findings, health target values were still less often met by women in terms of physical behaviour and psychosocial well-being, and by men in aerobic capacity. Tailored CR programmes that consider the unique needs of women and men might be warranted to address these gaps and to optimize outcomes for both sexes.

## Supplementary Material


