# Supplementary material for: WOMEN AND MEN PROFIT EQUALLY FROM CARDIAC REHABILITATION: A SECONDARY ANALYSIS OF THE OPTICARE RCT
Source: J Rehabil Med. 2026 Jan 7;58:44504. doi: 10.2340/jrm.v58.44504 (PMC12794302; doi:10.2340/jrm.v58.44504)
Supplement: Supplementary file 1 [file JRM-58-44504-s1.pdf]

Supplementary material has been published as submitted. It has not been copyedited, or typeset by Journal of Rehabilitation Medicine

Appendix 1 Comparison of Missing Data Between Women and Men

	Pre-CR			Post-CR			18 months follow-up		
	**Women ♀ (n=147)**	**Men ♂ (n=624)**	**P-value***	**Women ♀ (n=147)**	**Men ♂ (n=624)**	**P-value***	**Women♀ (n=147)**	**Men ♂ (n=624)**	**P-value***
6-Minute Walk Test	37 (25.2)	136 (21.8)	0.378	50 (34.0)	199 (31.9)	0.621	53 (36.1)	221 (35.4)	0.884
Physical behaviour	63 (42.9)	242(38.8)	0.363	73 (49.7)	299(47.9)	0.704	79 (53.7)	333(53.4)	0.934
Body mass index	0	1 (0.2)	0.809	9 (6.1)	20 (3.2)	0.094	27 (18.4)	115 (18.4)	0.986
Systolic BP	0	0	-	10 (6.8)	31 (5.0)	0.372	16 (10.9)	83 (13.3)	0.431
LDL cholesterol	17 (11.6)	48 (7.7)	0.128	31 (21.1)	98 (15.7)	0.116	16 (10.9)	93 (14.9)	0.208
HDL cholesterol	13 (8.8)	28 (4.5)	**0.034**	30 (20.4)	95 (15.2)	0.125	16 (10.9)	92 (14.7)	0.225
Smoking	6 (4.1)	12 (1.9)	0.119	28 (19.0)	86 (13.8)	0.106	27 (18.4)	134 (21.5)	0.404
Anxiety symptoms	27 (18.4)	127 (20.4)	0.588	43 (29.3)	148 (23.7)	0.162	49 (33.3)	191 (30.6)	0.521
Depressive symptoms	29 (19.7)	129 (20.7)	0.798	43 (29.3)	147 (23.6)	0.150	49 (33.3)	194 (31.1)	0.598
Participation in Society	31 (21.1)	125 (20.0)	0.774	41 (27.9)	140 (22.4)	0.160	50 (34.0)	198 (31.7)	0.594
HRQOL	29 (19.7)	129 (20.7)	0.798	43 (29.3)	144 (23.1)	0.116	48 (32.7)	196 (31.4)	0.771

BP= Blood Pressure; LDL= Low Density Lipoprotein cholesterol; HDL= High Density Lipoprotein Cholesterol; HRQOL=Health Related Quality of Life

**P-values based on Chi-Square tests or Fisher's Exact tests.*

Appendix 2 Sex differences in CR outcomes

Appendix 2a. Sex differences in aerobic capacity and physical behaviour outcomes

Outcome	Comparison	Women ♀ Adjusted mean change* (95%CI)	Women ♀ P-value	Men ♂ Adjusted mean change* (95%CI)	Men ♂ P-value	P-value ♂ *vs.* ♀
**6-Minute Walk Test (m)** *(n=728)*	Adjusted baseline	512.0 (443.2;580.8)		571.9 (501.8;642.0)		<0.001
	Post-CR vs. pre-CR	33.7 (21.8;45.5)	<0.001	40.0 (33.2;46.7)	<0.001	0.364
	18-months follow-up vs. post-CR	-9.2 (-21.6;3.1)	0.144	-1.5 (-7.8;4.9)	0.656	0.273
**Steps per day** *(n=541)*	Adjusted baseline	5590.7 (3542.4;7639.1)		6123.9 (4003.8;8244.1)		0.023
	Post-CR vs. pre-CR	876.5 (504.4;1248.7)	<0.001	922.9 (690.4;1155.4)	<0.001	0.836
	18-months follow-up vs. post-CR	-229.0 (-656.6;198.6)	0.294	-194.9 (-471.1;81.3)	0.167	0.895
**Time in Moderate to Vigorous Physical Activity (%)** (*n=541)*	Adjusted baseline	4.8 (2.3;7.3)		6.2 (3.6;8.8)		<0.001
	Post-CR vs. pre-CR	1.1 (0.7;1.6)	<0.001	0.9 (0.6;1.1)	<0.001	0.324
	18-months follow-up vs. post-CR	-0.3 (-0.8;0.3)	0.352	-0.2 (-0.5;0.1)	0.191	0.875
**Time in Light Activity (%)** (*n=541)*	Adjusted baseline	31.2 (24.4;38.0)		28.1 (21.5;34.8)		<0.001
	Post-CR vs. pre-CR	1.6 (0.5;2.7)	0.005	1.5 (0.8;2.1)	<0.001	0.849
	18-months follow-up vs. post-CR	-0.1 (-1.5;1.2)	0.844	0.1 (-0.6;0.9)	0.741	0.742
**Time in Sedentary Behaviour (%)** (*n=541)*	Adjusted baseline	64.0 (56.3;71.7)		65.7 (58.0;73.3)		0.059
	Post-CR vs. pre-CR	-2.7 (-4.0;-1.5)	<0.001	-2.3 (-3.1;-1.6)	<0.001	0.590
	18-months follow-up vs. post-CR	0.4 (-1.2;1.9)	0.618	0.1 (-0.8;1.0)	0.836	0.740

CR= cardiac rehabilitation

**adjusted for confounding effect of age, marital status, employment, medical treatment and BMI.*

Appendix 2b. Sex differences in cardiovascular risk profile

Outcome	Comparison	Women ♀ Adjusted mean change (95%CI)	P-value	Men ♂ Adjusted mean change (95%CI)	P-value	P-value ♂ *vs.* ♀
**Body mass index *** *(n=770)*	Adjusted baseline	27.6 (24.8;30.4)		28.1 (25.4;30.8)		0.225
	Post-CR vs. pre-CR	0.3 (0.01;0.7)	0.046	-0.2 (-0.3;-0.1)	<0.001	0.001
	18-months follow-up vs. post-CR	-0.1 (-0.5;0.3)	0.639	0.3 (0.2;0.5)	<0.001	0.048
**Systolic blood pressure (mmHg) **** *(n=771)*	Adjusted baseline	132.9 (120.9;145.0)		130.4 (118.5;142.2)		0.130
	Post-CR vs. pre-CR	-7.5 (-10.7;-4.4)	<0.001	-4.2 (-5.7;-2.8)	<0.001	0.063
	18-months follow-up vs. post-CR	9.1 (5.9;12.4)	<0.001	6.6 (5.0;8.1)	<0.001	0.166
**LDL cholesterol (mmol/L) **** (*n=760)*	Adjusted baseline	2.5 (1.9;3.2)		2.3 (1.7;3.0)		0.029
	Post-CR vs. pre-CR	0.01 (-0.1;0.1)	0.934	-0.01 (-0.07;0.04)	0.612	0.783
	18-months follow-up vs. post-CR	-0.03 (-0.2;0.1)	0.718	0.06 (0;0.1)	0.063	0.259
**HDL cholesterol (mmol/L) **** (*n=761)*	Adjusted baseline	1.3 (1.1;1.6)		1.1 (0.9;1.4)		<0.001
	Post-CR vs. pre-CR	0.01 (-0.03;0.04)	0.689	0.04 (0.02;0.05)	<0.001	0.077
	18-months follow-up vs. post-CR	0.04 (0.01;0.08)	0.019	0.04 (0.02;0.06)	<0.001	0.891
		**Odds ratio (95%CI)**	**P-value**	**Odds ratio (95%CI)**	**P-value**	**P-value ♂ *vs.* ♀**
**Smoker**** (*n=771)*	Adjusted baseline					0.541
	Post-CR vs. pre-CR	1.2 (0.8;1.7)	0.337	1.2 (1.0;1.5)	0.094	0.993
	18-months follow-up vs. post-CR	1.7 (1.1;2.6)	0.012	1.7 (1.3;2.2)	<0.001	0.951

CR= cardiac rehabilitation

**adjusted for confounding effect of age, marital status, employment and medical treatment.*

***adjusted for confounding effect of age, marital status, employment, medical treatment and BMI.*

Appendix 2c. Sex differences in psychosocial well-being

Outcome	Comparison	Women ♀ Adjusted mean change* (95%CI)	Women ♀ P-value	Men ♂ Adjusted mean change* (95%CI)	Men ♂ P-value	P-value ♂ *vs.* ♀
**Anxiety symptoms** *(n=712)*	Adjusted baseline	7.3 (4.0;10.6)		4.8 (1.5;8.1)		<0.001
	Post-CR vs. pre-CR	-1.2 (-1.8;-0.6)	<0.001	-0.6 (-0.8;-0.3)	<0.001	0.083
	18-months follow-up vs. post-CR	-0.5 (-1.1;0.2)	0.163	-0.1 (-0.4;0.1)	0.374	0.344
**Depressive symptoms** *(n=713)*	Adjusted baseline	4.9 (1.7;8.1)		3.4 (0.2;6.7)		0.001
	Post-CR vs. pre-CR	-1.4 (-2.0;-0.8)	<0.001	-0.6 (-0.8;-0.3)	<0.001	0.009
	18-months follow-up vs. post-CR	0.1 (-0.5;0.6)	0.734	-0.1 (-0.4;0.2)	0.541	0.562
**Participation in society (satisfaction)** (*n=710)*	Adjusted baseline	63.7 (50.6;76.7)		68.5 (55.4;81.6)		0.002
	Post-CR vs. pre-CR	6.2 (3.6;8.9)	<0.001	5.7 (4.4;7.1)	<0.001	0.738
	18-months follow-up vs. post-CR	1.7 (-0.7;4.6)	0.141	-0.5 (-1.7;0.8)	0.475	0.102
**HRQOL** *(n=713)*	Adjusted baseline	4.7 (3.8;5.6)		5.3 (4.4;6.2)		<0.001
	Post-CR vs. pre-CR	0.6 (0.4;0.8)	<0.001	0.4 (0.4;0.5)	<0.001	0.119
	18-months follow-up vs. post-CR	0.2 (0.1;0.4)	0.003	0.2 (0.1;0.3)	<0.001	0.643

CR= cardiac rehabilitation; HRQOL= Health related Quality of Life
**adjusted for confounding effect of age, marital status, employment, medical treatment and BMI.*

Appendix 3 Secondary analysis: Sex differences in patients randomized to standard CR (CR-only)

Appendix 3a. Sex differences in aerobic capacity and physical behaviour outcomes in patients randomized to standard CR (CR-only)

Outcome	Comparison	Women ♀ Adjusted mean change* (95%CI)	Women ♀ P-value	Men ♂ Adjusted mean change* (95%CI)	Men ♂ P-value	P-value ♂ *vs.* ♀
**6-Minute Walk Test (m)** *(n=238)*	Baseline	507.7 (390.2;625.1)		569.7 (452.7;686.7)		<0.001
	Post-CR vs. pre-CR	34.4 (17.3;51.6)	<0.001	39.0 (26.0;52.0)	<0.001	0.680
	18-months follow-up vs. post-CR	-6.8 (-23.7;10.1)	0.431	2.5 (-9.6;14.6)	0.685	0.379
**Steps per day** *(n=174)*	Baseline	5682.9 (2197.6;9168.4)		6318.1 (2673.8;9962.4)		0.117
	Post-CR vs. pre-CR	1026.4 (417.2;1635.6)	0.001	704.1 (278.5;1129.6)	0.001	0.396
	18-months follow-up vs. post-CR	-825.4 (-1397.9;-253.0)	0.005	-270.4 (-738.7;197.8)	0.258	0.141
**Time in Moderate to Vigorous Physical Activity (%)** (*n=174)*	Baseline	5.0 (0.9;9.1)		6.7 (2.5;10.9)		<0.001
	Post-CR vs. pre-CR	1.5 (0.8;2.2)	<0.001	0.5 (0.05;1.0)	0.030	0.024
	18-months follow-up vs. post-CR	-0.4 (-1.2;0.4)	0.289	-0.3 (-0.8;0.2)	0.269	0.786
**Time in Light Activity (%)** (*n=174)*	Baseline	31.1 (19.4;42.8)		27.7 (16.2;39.1)		0.010
	Post-CR vs. pre-CR	2.7 (0.9;4.4)	0.003	1.9 (0.8;3.1)	0.001	0.495
	18-months follow-up vs. post-CR	-0.7 (-3.0;1.5)	0.533	0.2 (-1.1;1.5)	0.794	0.500
**Time in Sedentary Behaviour (%)** (*n=174)*	Baseline	63.9 (50.6;77.1)		65.6 (52.5;78.7)		0.233
	Post-CR vs. pre-CR	-4.2 (-6.2;-2.1)	<0.001	-2.4 (-3.8;-1.1)	<0.001	0.167
	18-months follow-up vs. post-CR	1.1 (-1.3;3.5)	0.366	0.1 (-1.4;1.7)	0.861	0.501

CR= cardiac rehabilitation
**adjusted for confounding effect of age, marital status, employment, therapeutic intervention and BMI.*

Appendix 3b: Sex differences in cardiovascular risk profile in patients randomized to standard CR (CR-only)

		Women ♀		Men ♂		
**Outcome**	**Comparison**	**Adjusted mean change (95%CI)**	**P-value**	**Adjusted mean change (95%CI)**	**P-value**	**P-value ♂ *vs.* ♀**
**Body mass index *** *(n=248)*	Baseline	28.0 (23.3;32.7)		28.0 (23.5;32.5)		0.987
	Post-CR vs. pre-CR	0.2 (-0.1;0.4)	0.319	-0.3 (-0.5;-0.1)	0.001	0.007
	18-months follow-up vs. post-CR	0.4 (-0.2;0.9)	0.161	0.4 (0.1;0.6)	0.002	0.978
**Systolic blood pressure (mmHg) **** *(n=248)*	Baseline	135.3 (115.7;154.9)		131.2 (112.4;149.9)		0.147
	Post-CR vs. pre-CR	-10.3 (-15.5;-5.0)	<0.001	-4.2 (-6.8;-1.6)	0.002	0.042
	18-months follow-up vs. post-CR	8.4 (3.3;13.6)	0.001	6.4 (3.5;9.2)	<0.001	0.495
**LDL cholesterol (mmol/L) **** (*n=246)*	Baseline	2.7 (1.6;3.9)		2.3 (1.1;3.4)		0.005
	Post-CR vs. pre-CR	0.01 (-0.3;0.3)	0.955	0.1 (-0.02;0.2)	0.107	0.620
	18-months follow-up vs. post-CR	-0.05 (-0.3;0.2)	0.604	0.1 (-0.01;0.2)	0.066	0.164
**HDL cholesterol (mmol/L) **** (*n=246)*	Baseline	1.3 (0.9;1.8)		1.1 (0.7;1.5)		<0.001
	Post-CR vs. pre-CR	0.00 (-0.06;0.05)	0.924	0.02 (0.00;0.04)	0.059	0.441
	18-months follow-up vs. post-CR	0.07 (0.0;0.1)	0.052	0.04 (0.01;0.06)	0.007	0.356
		**Odds ratio (95%CI)**	**P-value**	**Odds ratio (95%CI)**	**P-value**	**P-value ♂ *vs.* ♀**
**Smoker**** (*n=248)*	Baseline					0.362
	Post-CR vs. pre-CR	1.0 (0.9;1.1)	0.589	1.06 (1.0;1.1)	0.006	0.104
	18-months follow-up vs. post-CR	1.2 (1.0;1.4)	0.013	1.05 (1.0;1.1)	0.069	0.107

CR= cardiac rehabilitation
**adjusted for confounding effect of age, marital status, employment, therapeutic intervention and BMI.*
***adjusted for confounding effect of age, marital status, employment and therapeutic intervention.*

Appendix 3c. Sex differences in psychosocial well-being in patients randomized to standard CR (CR-only)

Outcome	Comparison	Women ♀ Adjusted mean change* (95%CI)	Women ♀ P-value	Men ♂ Adjusted mean change* (95%CI)	Men ♂ P-value	P-value ♂ *vs.* ♀
**Anxiety symptoms** *(n=231)*	Adjusted baseline	7.3 (1.4;13.1)		5.0 (-0.8;10.9)		0.005
	Post-CR vs. pre-CR	-0.6 (-1.5;0.3)	0.197	-0.8 (-1.2;-0.4)	<0.001	0.674
	18-months follow-up vs. post-CR	-0.6 (-2.1;0.9)	0.432	0.0 (-0.4;0.5)	0.829	0.415
**Depressive symptoms** *(n=232)*	Adjusted baseline	4.1 (-1.5;9.8)		3.9 (-1.8;9.5)		0.726
	Post-CR vs. pre-CR	-0.2 (-0.9;0.5)	0.602	-0.7 (-1.1;-0.3)	0.001	0.192
	18-months follow-up vs. post-CR	-0.5 (-1.7;0.6)	0.345	0.0 (-0.5;0.5)	0.929	0.368
**Participation in society (satisfaction)** (*n=231)*	Baseline	65.8 (43.8;87.9)		68.7 (46.8;90.7)		0.290
	Post-CR vs. pre-CR	3.6 (-0.4;7.7)	0.077	4.8 (2.7;6.8)	<0.001	0.625
	18-months follow-up vs. post-CR	1.4 (-3.0;5.8)	0.521	-0.4 (-2.5;1.8)	0.737	0.469
**HRQOL** *(n=232)*	Baseline	4.9 (3.1;6.6)		5.2 (3.5;6.9)		0.112
	Post-CR vs. pre-CR	0.4 (0.1;0.6)	0.010	0.5 (0.4;0.6)	<0.001	0.440
	18-months follow-up vs. post-CR	0.2 (-0.1;0.6)	0.099	0.2 (0.1;0.4)	0.001	0.851

CR= cardiac rehabilitation; HRQOL= Health related Quality of Life
**adjusted for confounding effect of age, marital status, employment, therapeutic intervention and BMI.*

Appendix 4 Secondary analysis: sex differences in patients randomized to CR with additional face-to-face coaching (CR+F)

Appendix 4a. Sex differences in aerobic capacity and physical behaviour outcomes in patients randomized to CR with additional face-to-face coaching (CR+F)

Outcome	Comparison	Women ♀ Adjusted mean change* (95%CI)	Women ♀ P-value	Men ♂ Adjusted mean change* (95%CI)	Men ♂ P-value	P-value ♂ *vs.* ♀	P-value sex differences CR+F vs CR-only[1]
**6-Minute Walk Test (m)** *(n=255)*	Baseline	505.7 (389.2;622.1)		572.6 (455.6;689.6)		<0.001	
	Post-CR vs. pre-CR	43.8 (22.7;64.8)	<0.001	43.9 (34.3;53.5)	<0.001	0.994	0.820
	18-months follow-up vs. post-CR	-11.0 (-33.6;11.6)	0.338	-4.7 (-14.9;5.5)	0.365	0.617	0.932
**Steps per day** *(n=187)*	Baseline	5602.6 (2278.5;8926.7)		6102.5 (2813.2;9391.9)		0.173	
	Post-CR vs. pre-CR	995.5 (378.2;1612.8)	0.002	1551.6 (1157.0;1946.2)	<0.001	0.138	0.106
	18-months follow-up vs. post-CR	-107.4 (-638.0;423.3)	0.692	-447.6 (-995.1;99.9)	0.109	0.381	0.116
**Time in Moderate to Vigorous Physical Activity (%)** (*n=187)*	Baseline	4.8 (0.6;9.0)		6.0 (1.8;10.2)		0.012	
	Post-CR vs. pre-CR	1.0 (0.2;1.8)	0.011	1.5 (1.1;2.0)	<0.001	0.259	0.019
	18-months follow-up vs. post-CR	-0.6 (-1.2;-0.04)	0.037	-0.6 (-1.2;0.04)	0.065	0.937	0.912
**Time in Light Activity (%)** (*n=187)*	Baseline	31.2 (19.1;43.4)		28.3 (16.5;40.2)		0.022	
	Post-CR vs. pre-CR	0.1 (-1.9;2.1)	0.920	0.9 (-0.1;1.8)	0.094	0.506	0.407
	18-months follow-up vs. post-CR	1.9 (-0.4;4.3)	0.107	0.4 (-0.7;1.5)	0.458	0.255	0.274
**Time in Sedentary Behaviour (%)** (*n=187)*	Baseline	64.0 (50.2;77.8)		65.7 (52.1;79.3)		0.230	
	Post-CR vs. pre-CR	-1.2 (-3.2;0.8)	0.254	-2.4 (-3.6;-1.2)	<0.001	0.286	0.104
	18-months follow-up vs. post-CR	-1.3 (-3.9;1.3)	0.317	0.2 (-1.3;1.6)	0.803	0.320	0.321

CR= cardiac rehabilitation

**adjusted for confounding effect of age, marital status, employment, therapeutic intervention and BMI.*

[1]*The difference in mean change between men and women in the CR-only group was compared to the difference in mean change between men and women in the CR+F group using a Z-test.*

Appendix 4b: Sex differences in cardiovascular risk profile in patients randomized to CR with additional face-to-face coaching (CR+F)

Outcome	Comparison	Women ♂ Adjusted mean change (95%CI)	Women ♂ P-value	Men ♀ Adjusted mean change (95%CI)	Men ♀ P-value	P-value ♂ *vs.* ♀	P-value sex differences CR+F vs CR-only[1]
**Body mass index *** *(n=267)*	Baseline	27.3 (22.1;32.4)		28.2 (23.4;33.0)		0.191	
	Post-CR vs. pre-CR	0.4 (-0.3;1.2)	0.227	-0.1 (-0.3;0.1)	0.208	0.135	0.841
	18-months follow-up vs. post-CR	-0.5 (-1.4;0.4)	0.244	0.4 (0.2;0.7)	<0.001	0.035	0.080
**Systolic blood pressure (mmHg) **** *(n=268)*	Baseline	133.4 (112.8;154.0)		130.7 (110.5;151.0)		0.303	
	Post-CR vs. pre-CR	-6.9 (-11.6;-2.2)	0.004	-4.7 (-7.2;-2.2)	<0.001	0.422	0.282
	18-months follow-up vs. post-CR	9.5 (3.5;15.5)	0.002	5.6 (2.7;8.5)	<0.001	0.244	0.738
**LDL cholesterol (mmol/L) **** (*n=263)*	Baseline	2.4 (1.1;3.7)		2.4 (1.2;3.6)		1.000	
	Post-CR vs. pre-CR	-0.1 (-0.3;0.1)	0.298	-0.2 (-0.3;-0.1)	0.001	0.652	0.501
	18-months follow-up vs. post-CR	0.05 (-0.2;0.3)	0.704	0.04 (-0.1;0.2)	0.536	0.944	0.334
**HDL cholesterol (mmol/L) **** (*n=264)*	Baseline	1.3 (0.8;1.8)		1.1 (0.6;1.6)		<0.001	
	Post-CR vs. pre-CR	0.02 (-0.04;0.07)	0.603	0.04 (0.02;0.07)	<0.001	0.376	0.890
	18-months follow-up vs. post-CR	0.02 (-0.02;0.07)	0.305	0.03 (0.00;0.07)	0.085	0.762	0.384
		**Odds ratio (95%CI)**	**P-value**	**Odds ratio (95%CI)**	**P-value**	**P-value ♂ *vs.* ♀**	
**Smoker**** *(n=268)*	Baseline					0.818	
	Post-CR vs. pre-CR	1.04 (0.97;1.1)	0.306	1.02 (0.98;1.06)	0.418	0.629	0.128
	18-months follow-up vs. post-CR	1.03 (0.95;1.1)	0.427	1.1 (1.0;1.2)	0.020	0.422	0.174

CR= cardiac rehabilitation

**adjusted for confounding effect of age, marital status, employment, therapeutic intervention and BMI.*

***adjusted for confounding effect of age, marital status, employment and therapeutic intervention.*

[1]*The difference in mean change between men and women in the CR-only group was compared to the difference in mean change between men and women in the CR+F group using a Z-test.*

Appendix 4c. Sex differences in psychosocial well-being in patients randomized to CR with additional face-to-face coaching (CR+F)

Outcome	Comparison	Women ♂ Adjusted mean change* (95%CI)	Women ♂ P-value	Men ♀ Adjusted mean change* (95%CI)	Men ♀ P-value	P-value ♂ *vs.* ♀	P-value sex differences CR+F vs CR-only[1]
**Anxiety symptoms** *(n=245)*	Baseline	7.2 (1.7;12.8)		4.7 (-0.8;10.1)		<0.001	
	Post-CR vs. pre-CR	-1.3 (-2.4;-0.1)	0.030	-0.6 (-1;-0.2)	0.007	0.265	0.244
	18-months follow-up vs. post-CR	-0.5 (-1.4;0.4)	0.293	0.0 (-0.6;0.5)	0.921	0.399	0.886
**Depressive symptoms** *(n =245)*	Baseline	4.6 (-0.3;9.4)		3.5 (-1.2;8.3)		0.116	
	Post-CR vs. pre-CR	-1.8 (-2.7;-0.9)	<0.001	-0.6 (-1.0;-0.1)	0.017	0.017	0.005
	18-months follow-up vs. post-CR	0.2 (-0.5;0.9)	0.550	-0.2 (-0.7;0.4)	0.497	0.375	0.201
**Participation in society (satisfaction)** *(n=245)*	Baseline	63.4 (39.0;87.8)		67.9 (43.7;92.0)		0.069	
	Post-CR vs. pre-CR	5.4 (1.7;9.1)	0.005	6.1 (3.5;8.7)	<0.001	0.739	0.872
	18-months follow-up vs. post-CR	1.1 (-2.6;4.9)	0.551	-0.5 (-2.9;1.9)	0.666	0.462	0.846
**HRQOL** *(n=245)*	Baseline	4.6 (3.1;6.1)		5.3 (3.9;6.8)		<0.001	
	Post-CR vs. pre-CR	0.6 (0.4;0.9)	<0.001	0.4 (0.3;0.6)	<0.001	0.240	0.138
	18-months follow-up vs. post-CR	0.2 (-0.01;0.5)	0.057	0.2 (0.1;0.3)	0.005	0.875	0.831

CR= cardiac rehabilitation; HRQOL= Health related Quality of Life
**adjusted for confounding effect of age, marital status, employment, therapeutic intervention and BMI.*
[1]*The difference in mean change between men and women in the CR-only group was compared to the difference in mean change between men and women in the CR+F group using a Z-test.*

Appendix 5 Secondary analysis: sex differences in patients randomized to CR with additional coaching by telephone (CR+T)

Appendix 5a. Sex differences in aerobic capacity and physical behaviour outcomes in patients randomized to CR with additional coaching by telephone (CR+T)

Outcome	Comparison	Women ♂<br>Adjusted mean change* (95%CI)	Women ♂<br>P-value	Men ♀<br>Adjusted mean change* (95%CI)	Men ♀<br>P-value	P-value ♂ *vs.* ♀	P- value sex differences CR+T vs CR-only[1]
**6-Minute Walk Test (m)** *(n=235)*	Baseline	520.8 (390.6;651.0)		574.0 (439.0;709.0)		<0.001	
	Post-CR vs. pre-CR	20.4 (-3.7;44.5)	0.097	37.0 (24.9;49.2)	<0.001	0.227	0.468
	18-months follow-up vs. post-CR	-8.6 (-33.5;16.3)	0.500	-1.9 (-12.7;9.0)	0.737	0.627	0.893
**Steps per day** *(n=180)*	Baseline	5403.4 (1715.8;9091.0)		5986.5 (2131.3;9841.6)		0.185	
	Post-CR vs. pre-CR	518.6 (-192.3;1229.4)	0.153	551.7 (189.6;913.9)	0.003	0.935	0.595
	18-months follow-up vs. post-CR	354.3 (-822.0;1530.6)	0.555	157.1 (-238.1;552.4)	0.436	0.755	0.287
**Time in Moderate to Vigorous Physical Activity (%)** (*n=180)*	Baseline	4.6 (0.2;9.1)		6.0 (1.2;10.8)		0.011	
	Post-CR vs. pre-CR	0.8 (-0.1;1.6)	0.076	0.6 (0.1;1.01)	0.022	0.665	0.286
	18-months follow-up vs. post-CR	0.5 (-1.0;2.1)	0.498	0.3 (-0.2;0.8)	0.295	0.748	0.539
**Time in Light Activity (%)** (*n=180)*	Baseline	31.2 (19.3;43.1)		28.4 (16.6;40.2)		0.041	
	Post-CR vs. pre-CR	2.3 (0.3;4.3)	0.024	1.6 (0.4;2.7)	0.011	0.519	0.918
	18-months follow-up vs. post-CR	-2.2 (-4.6;0.2)	0.076	-0.3 (-1.6;1.0)	0.682	0.174	0.568
**Time in Sedentary Behaviour (%)** (*n=180)*	Baseline	64.0 (50.8;77.3)		65.6 (52.2;79.0)		0.350	
	Post-CR vs. pre-CR	-3.0 (-5.5;-0.4)	0.023	-2.1 (-3.5;-0.7)	0.003	0.558	0.731
	18-months follow-up vs. post-CR	1.7 (-1.5;4.9)	0.296	0.02 (-1.5;1.6)	0.978	0.354	0.781

CR= cardiac rehabilitation
**adjusted for confounding effect of age, marital status, employment, therapeutic intervention and BMI.*

Appendix 5b: Sex differences in cardiovascular risk profile in patients randomized to CR with additional coaching by telephone (CR+T)

Outcome	Comparison	Women ♂ Adjusted mean change (95%CI)	Women ♂ P-value	Men ♀ Adjusted mean change (95%CI)	Men ♀ P-value	P-value ♂ *vs.* ♀	P- value sex differences CR+T vs CR-only[1]
**Body mass index *** *(n=255)*	Baseline	27.4 (23.0;31.8)		28.0 (23.7;32.3)		0.467	
	Post-CR vs. pre-CR	0.4 (-0.1;0.9)	0.150	-0.2 (-0.4;-0.04)	0.020	0.031	0.655
	18-months follow-up vs. post-CR	-0.2 (-0.8;0.5)	0.609	0.2 (-0.04;0.4)	0.100	0.314	0.464
**Systolic blood pressure (mmHg) **** *(n=255)*	Baseline	129.7 (108.7;150.7)		129.3 (108.1;150.5)		0.895	
	Post-CR vs. pre-CR	-5.5 (-11.7;0.8)	0.086	-3.8 (-6.5;-1.2)	0.004	0.639	0.294
	18-months follow-up vs. post-CR	8.9 (3.5;14.3)	0.001	7.6 (5.1;10.1)	<0.001	0.669	0.839
**LDL cholesterol (mmol/L) **** (*n=251)*	Baseline	2.5 (1.4;3.6)		2.3 (1.2;3.5)		0.277	
	Post-CR vs. pre-CR	0.2 (-0.03;0.4)	0.096	0.06 (-0.04;0.2)	0.251	0.305	0.304
	18-months follow-up vs. post-CR	-0.1 (-0.4;0.2)	0.384	0.04 (-0.06;0.1)	0.451	0.282	0.983
**HDL cholesterol (mmol/L) **** (*n=251)*	Baseline	1.3 (0.9;1.8)		1.1 (0.7;1.6)		<0.001	
	Post-CR vs. pre-CR	0.01 (-0.04;0.06)	0.803	0.05 (0.03;0.08)	<0.001	0.125	0.600
	18-months follow-up vs. post-CR	0.03 (-0.02;0.09)	0.252	0.05 (0.02;0.07)	<0.001	0.687	0.375
		**Odds ratio (95%CI)**	**P-value**	**Odds ratio (95%CI)**	**P-value**	**P-value ♂ *vs.* ♀**	
**Smoker**** *(n=255)*	Baseline					0.756	
	Post-CR vs. pre-CR	1.05 (0.96; 1.15)	0.250	0.98 (0.95; 1.02)	0.380	0.161	0.031
	18-months follow-up vs. post-CR	1.05 (0.96;1.15)	0.268	1.09 (1.04;1.15)	<0.001	0.443	0.091

CR= cardiac rehabilitation

**adjusted for confounding effect of age, marital status, employment, therapeutic intervention and BMI.*

***adjusted for confounding effect of age, marital status, employment and therapeutic intervention.*

Appendix 5c. Sex differences in psychosocial well-being in patients randomized to CR with additional coaching by telephone (CR+T)

Outcome	Comparison	**Women ♂** Adjusted mean change* (95%CI)	**Women ♂** P-value	**Men ♀** Adjusted mean change* (95%CI)	**Men ♀** P-value	P-value ♂ *vs.* ♀	P- value sex differences CR+T vs CR-only[1]
**Anxiety symptoms** *(n=236)*	Baseline	7.5 (3.2;11.9)		4.7 (0.1;9.3)		0.001	
	Post-CR vs. pre-CR	-1.8 (-3;-0.5)	0.007	-0.4 (-0.8;0.1)	0.088	0.047	0.067
	18-months follow-up vs. post-CR	-0.2 (-1.1;0.8)	0.743	-0.5 (-0.9;-0.1)	0.024	0.566	0.319
**Depressive symptoms** *(n =236)*	Baseline	6.2 (1.6;10.7)		3.0 (-1.6;7.6)		<0.001	
	Post-CR vs. pre-CR	-2.4 (-3.7;-1.0)	0.001	-0.3 (-0.7;0.1)	0.090	0.005	0.002
	18-months follow-up vs. post-CR	0.7 (-0.4;1.7)	0.221	-0.3 (-0.7;0.1)	0.125	0.097	0.077
**Participation in society (satisfaction)** (*n=234)*	Baseline	61.0 (39.6;82.4)		69.0 (46.9;91.1)		0.006	
	Post-CR vs. pre-CR	9.7 (4.0;15.4)	0.001	6.2 (3.8;8.6)	<0.001	0.265	0.227
	18-months follow-up vs. post-CR	4.5 (-0.7;9.7)	0.092	-0.4 (-2.4;1.6)	0.728	0.090	0.490
**HRQOL** *(n=236)*	Baseline	4.6 (3.2;6,0)		5.4 (4.0;6.8)		<0.001	
	Post-CR vs. pre-CR	0.8 (0.5;1.1)	<0.001	0.4 (0.3;0.5)	<0.001	0.029	0.032
	18-months follow-up vs. post-CR	0.1 (-0.1;0.4)	0.308	0.2 (0.1;0.3)	<0.001	0.663	0.633

CR= cardiac rehabilitation; HRQOL= Health related Quality of Life
**adjusted for confounding effect of age, marital status, employment, therapeutic intervention and BMI.*
